# Investigation of the Effect of Blended Aggregate on the Strength and Drying Shrinkage Characteristics of Alkali-Activated Slag Mortar

**DOI:** 10.3390/ma17102211

**Published:** 2024-05-08

**Authors:** Choonghyun Kang, Yongmyung Park, Taewan Kim

**Affiliations:** 1 Department of Ocean Civil Engineering, Gyeongsang National University, Tongyeong 53064, Republic of Korea; chkang@gnu.ac.kr; 2 Department of Civil Engineering, Pusan National University, Busan 46241, Republic of Korea; ympk@pusan.ac.kr

**Keywords:** silica sand, river sand, mixed aggregate, drying shrinkage, alkali-activated slag

## Abstract

To reduce drying shrinkage of AASC mortar (AASM), mixed aggregate mixed with river sand (RS) and silica sand in three sizes was used to investigate the effect of the physical properties of mixed aggregate on shrinkage reduction. A mixture of river sand (0.2–0.8 mm), S1 (2.5–5.0 mm), S2 (1.6–2.5 mm), and S3 (1.21–160 mm) had river sand–silica sand mean diameter ratios (*dr*) of 7.68 (S1/RS), 3.75 (S2/RS), and 3.02 (S3/RS). The compressive strength and drying shrinkage characteristics of mixed aggregates according to fineness modulus, surface area, bulk density, and pore space were investigated. It had the highest bulk density and lowest porosity at a substitution ratio of 50%, but the highest strength was measured at a substitution ratio of 50% or less. High mechanical properties were shown when the fineness modulus of the mixed aggregate was in the range of 2.25–3.75 and the surface area was in the range of 2.25–4.25 m^2^/kg. As the substitution rate of silica sand increased, drying shrinkage decreased. In particular, the drying shrinkage of RS + S1 mixed aggregate mixed with S1 silica sand, which had the largest particle size, was the smallest. When silica sand or river sand was used alone, the drying shrinkage of the sample manufactured only with S1, which has the largest particle size of silica sand, was the smallest among all mixes. Compared to RS, at a 5% activator concentration, drying shrinkage was reduced by approximately 40% for S1, 27% for S2, and 19% for S3. At a 10% concentration, S1 showed a reduction effect of 39%, S2 by 28%, and S3 by 13%. As a result of this study, it was confirmed that the drying shrinkage of AASM could be reduced simply by controlling the physical properties of the aggregate mixed with two types of aggregate. This is believed to have a synergistic effect in reducing drying shrinkage when combined with various reduction methods published in previous studies on AASM shrinkage reduction. However, additional research is needed to analyze the correlation and influencing factors between the strength, pore structure, and drying shrinkage of AASM using mixed aggregate.

## 1. Introduction

Ordinary Portland cement (OPC) goes through a manufacturing process that consumes a lot of energy and emits carbon dioxide. The production of 1 ton of OPC emits about 0.94 tons of carbon dioxide, which is equivalent to about 6–7% of global greenhouse gases [1,2,3,4,5,6]. Moreover, recent global efforts to reduce greenhouse gases are also being required in the construction industry. Therefore, there is an urgent need to develop and distribute eco-friendly and sustainable cement. Among the many studies on eco-friendly cement, alkali-activated slag cement (AASC), which uses blast furnace slag generated in the steel industry as a precursor and an alkaline activator, is attracting attention [7,8,9,10]. It has been reported that AASC can reduce CO_2_ emissions by 25–80% relative to OPC [11,12,13]. AASC has similar or better mechanical performance and durability than OPC [14,15,16,17]. However, in order to apply AASC to full-scale construction, certain problems, such as low carbonation resistance [18,19,20], alkali–aggregate reaction [21], and shrinkage [22,23,24,25,26,27,28,29,30], must be solved or improved. According to previous studies, AAC exhibits a drying shrinkage rate that is about 2–5 times higher than that of OPC [31,32,33,34,35]. Recently, much attention has been focused on research on AAC contraction. This causes cracks in the concrete structure, which ultimately becomes a fatal weakness in the safety and durability of the structure.

Many researchers have conducted research to identify the complex and unclear contraction mechanism of AASC. It has been reported that the shrinkage behavior of AASC is affected by diverse and complex factors, such as humidity [32,36,37], the nature of raw materials [17,31,33], temperature [38,39], the drying history [40], the curing regime [41,42,43,44], pore size [33], the activator type and dosage [7,22,23,45,46,47], aggregate properties [48,49], and the exposure period [50]. Additionally, experiments and research have been conducted to study contraction prediction models for various AACs [27,34,51]. However, the contraction mechanism of AAC is still unclear, and control of contractions is difficult.

It is reported that the cause of the large drying shrinkage of AASC is due to the micropore structure [33,52,53] and the characteristics of the hydration reaction product gel [54,55]. As the pore structure becomes more dense, capillary pressure increases due to moisture loss [33]. In particular, AASC has a higher amount and distribution of micropores than OPC, which increases shrinkage due to a relatively high meniscus effect [35,36,56,57]. In addition, C(A)SH gel, the main hydration reactant of AASC, is known to cause viscous behavior and shrinkage because it has a lower Ca/Si ratio and a relatively shorter crystalline phase compared to Portland cement [36,40,58,59]. As a result, the shrinkage behavior of AASC is due to moisture loss due to a decrease in relative humidity (RH), and it is assumed that this is due to the combined effects of the pore shape and size distribution of the AASC matrix and the hydration reactant.

Various methods have been attempted to reduce the drying shrinkage of AAS. For example, the addition of fiber [60,61,62,63], pozzolanic material [57,58,59,60,61,62,63,64,65,66,67], an expansion agent [68,69,70], nano-materials [57,71,72,73], a shrinkage-reducing agent (SRA) [32,34,74,75,76,77], superabsorbent polymer [78,79], and methods for increasing the curing temperature [23,42,58,80,81] have been attempted. Previous studies on drying shrinkage reduction experiments and studies have shown a certain level of shrinkage reduction effect. However, most methods for reducing shrinkage have the following disadvantages: increased cost, need for additional equipment, addition of construction or manufacturing processes, low adaptability to changes in the field environment, and difficulty controlling the shrinkage rate to a certain level. These problems mean that more research and experimental data are still needed before an effective shrinkage reduction method is presented or a standard procedure is developed.

Most studies on the drying shrinkage of AASC published to date have focused on the binder’s constituent materials and proportions and exposure environmental conditions. However, studies on the drying shrinkage of AASC considering the influence of aggregates, such as mortar or concrete, are very rare. Chi et al. [82] noted that the drying shrinkage of AASC mortar could be controlled by increasing the aggregate to binder (A/B) ratio. They said that drying shrinkage was lowest when the A/B ratio was 2.0. A study by Adesanya et al. [83] also showed that the reduction in drying shrinkage was lowest when the volume fraction of the aggregate was about 60%. Chen et al. [84] published research results showing that it is effective in reducing drying shrinkage when the A/B ratio is more than 2.0 and the size of the aggregate is larger than 1.18 mm.

This study is an experiment that attempted a new approach to the role of the aggregate and its characteristics among studies to reduce the drying shrinkage of AASM. This experiment presents the possibility of and a method for reducing drying shrinkage by controlling the particle size characteristics of the aggregate, rather than the various methods applied in previous studies to reduce the drying shrinkage of AASC. The goal is to develop a method to reduce drying shrinkage without increasing time and cost due to the use of additional materials and mixing processes, which can be used as important data in the design and manufacturing of AASC concrete required for actual field application in the future. Based on previous research results, the drying shrinkage characteristics of AASM using mixed aggregate were examined by mixing river sand and silica sand of three different sizes in 10 different ratios. Based on the drying shrinkage characteristics of mixed aggregate, we will examine the drying shrinkage control effect of AASM by mixing aggregates of different particle sizes and use it as an important factor that can be considered when designing an AASM mix. This is expected to provide useful guidelines for shrinkage control in AAS concrete manufacturing and property studies applicable to actual sites.

## 2. Materials and Methods

### 2.1. Materials

**Figure 1:**
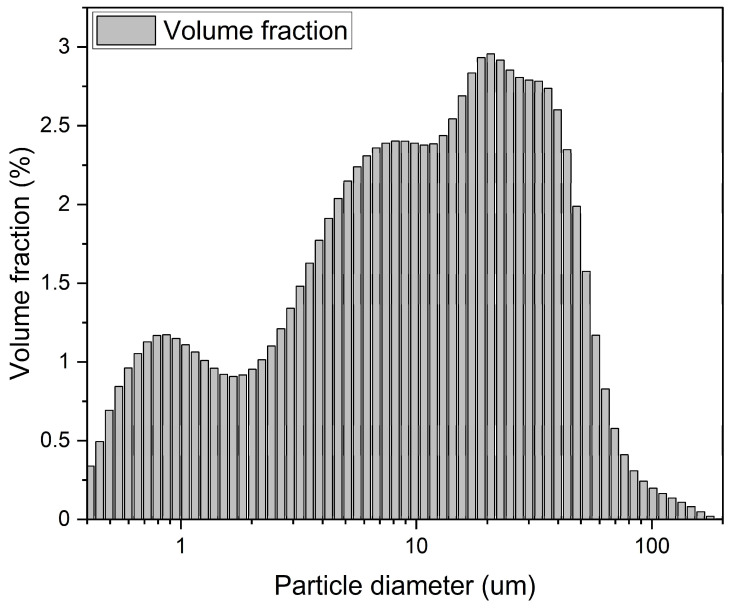
Slag particle size distribution curve.

The chemical composition of ground granulated blast furnace slag (slag, Maxcon Materials Co., Ltd., Boeun-gun, Republic of Korea), the main binder used in the production of AASC, is shown in Table 1. The slag used in this experiment had a density of 2.87 g/cm^3^, 4200 cm^2^/kg fineness, and 0.89% LOI. Figure 1 shows the analysis results using the Laser Diffraction Particle Size Analyzer (LS I3 320, Beckman Coulter, Indianapolis, IN, USA) equipment to analyze the particle size of slag particles. As a result of the analysis, the mean particle size of the slag particles was 18.10 mm, d_10_ = 1.105, d_50_ = 10.97, and d_90_ = 43.85 mm. The basicity of slag K_b_ = (CaO + MgO)/(SiO_2_ + Al_2_O_3_) is 2.43, and the hydraulic modulus H_m_ = (CaO + MgO + Al_2_O_3_)/SiO_2_ is 3.34. Sodium hydroxide (Samchun Chemical Co., Ltd., Seoul, Republic of Korea) and sodium silicate (Young Il Chemical Co., Ltd., Incheon, Republic of Korea) were used as activators, and two concentrations of 5% (5% NaOH + 5% Na_2_SiO_3_) and 10% (10% NaOH + 10% Na_2_SiO_3_) of the binder weight were considered, respectively. The alkaline activator was added to the mixing water at a predetermined concentration, stirred well, left to stand at room temperature for about 3 h, and then used for mixing mortar.

**Figure 2:**
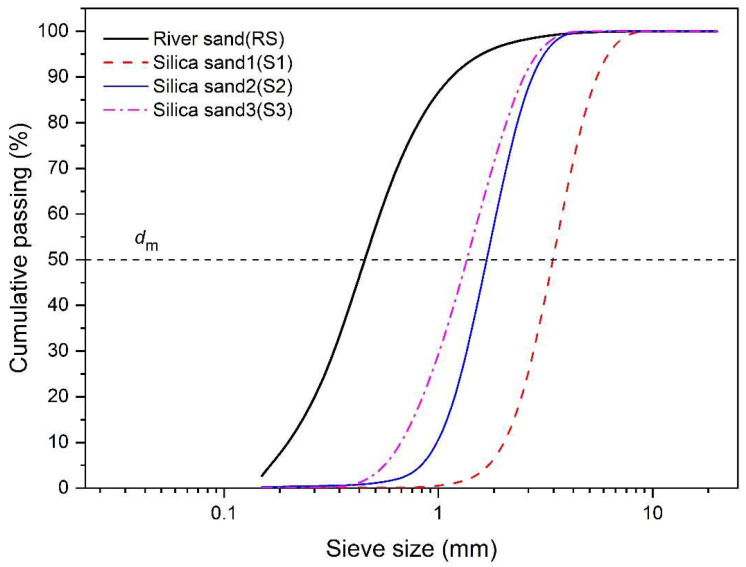
Particle size distribution of river sand and silica sands.

The fine aggregates used to manufacture mortar were river sand and silica sand (Gwang Myoung Material Co., Ltd., Incheon, Republic of Korea) of three types of sizes. The silica sand used in the experiment consisted of 97.8% SiO_2_, and it was made at the same production site; detailed specifications are shown in Table 2. Figure 2 shows particle size distribution curves obtained from sieving experiments performed according to the ASTM C136 [85] method for river sand and three types of silica sand. The bulk density (unit weight) and voids of the mixed aggregates were measured according to ASTM C29 [86]. In Table 2, the specific surface area is a value calculated using Equation (1), assuming that the particles of river sand and silica sand are spherical [87].

(1) $$\frac{6}{\rho d_{m}}$$

Here, *ρ* is the density of the fine aggregate and *d_m_* is the mean diameter of the aggregate.

The relative diameter ratio (*dr*) refers to the ratio of the average diameter of river sand and the average diameter of silica sand.

### 2.2. Methods

By investigating previous studies on the drying shrinkage of AASC mortar, it was reported that drying shrinkage tends to increase as the water–binder ratio (w/b) increases [88]. In this experiment, the effect of drying shrinkage was investigated using mixed fine aggregate in which river sand was partially replaced with silica sand. Previous studies have used various w/b to investigate the effect of drying shrinkage on AASC. In this study, preliminary experiments on various w/b were performed based on previous studies to determine the w/b. However, in this study, as silica sand, which has a larger particle size than river sand, was used, if w/b exceeded 0.4, molding of the test specimen became difficult, and material separation between the paste and the fine aggregate occurred. As a result, 0.4 was selected as the minimum w/b ratio that does not cause molding or material separation of the test specimen through preliminary experiments considering the conditions of this study and previous research [84] on the drying shrinkage of AASC specimens with different aggregate sizes. In studies on the drying shrinkage characteristics of AASC, reports on the influence of aggregates are very limited. However, when examining research results to date, it has been reported that when the aggregate to binder (A/B) ratio is 2.0, mechanical properties and drying shrinkage tend to be favorable [82,84]. Therefore, the A/B was selected as 2.0 in this study. No additional pozzolanic material or shrinkage-reducing agent (SRA) was used in the mix so as to only consider the effect of the aggregate. The purpose of this study was not to reduce drying shrinkage using various pozzolanic materials, water reducers, or shrinkage-reducing agents (SRAs) reported in previous studies of AASC but to examine the shrinkage reduction effect according to the characteristics of the aggregate. Therefore, pozzolanic materials, water reducers, and shrinkage-reducing agents (SRAs), which may affect the mechanical properties of AASC, hydration reactants, pore structure, and drying shrinkage, were not used.

The mixture of river sand and silica sand of three different sizes was replaced in 10% increments. The substitution of silica sand ultimately changes the fineness modulus and surface area of fine aggregate, which are important considerations for investigating the effect on shrinkage of AASC. The detailed mixing ratio is shown in Table 3.

A 50 × 50 × 50 mm^3^ cubic test specimen was used to measure the compressive strength, and the measured values of three samples are the average value. Samples for measuring drying shrinkage were manufactured on test specimens with a size of 25 × 25 × 285 mm^3^. Mortar mixing was conducted according to ASTM C305 [89].

The prepared mortar was poured into a mold and stored in a chamber at 23 ± 2 °C and a relative humidity of 90 ± 5%. After 1 day, the mold was removed, and the samples were stored in the chamber. Samples for measuring compressive strength were stored in a chamber at 23 ± 2 °C and a relative humidity of 90 ± 5% until the measurement date. Samples for drying shrinkage were stored in a chamber with a relative humidity of 50 ± 5% and 23 ± 2 °C in time for the start of measurement. The flow value of the AASC mortar was measured according to the ASTM C230 [90] method, and the compressive strength was measured according to ASTM C109 [91]. The drying shrinkage was measured according to ASTM C490 [92]. The porosity of the samples was measured according to the methods and procedures of ASTM C642 [93].

## 3. Results and Discussion

### 3.1. Properties of Mixed Aggregates

**Figure 3:**
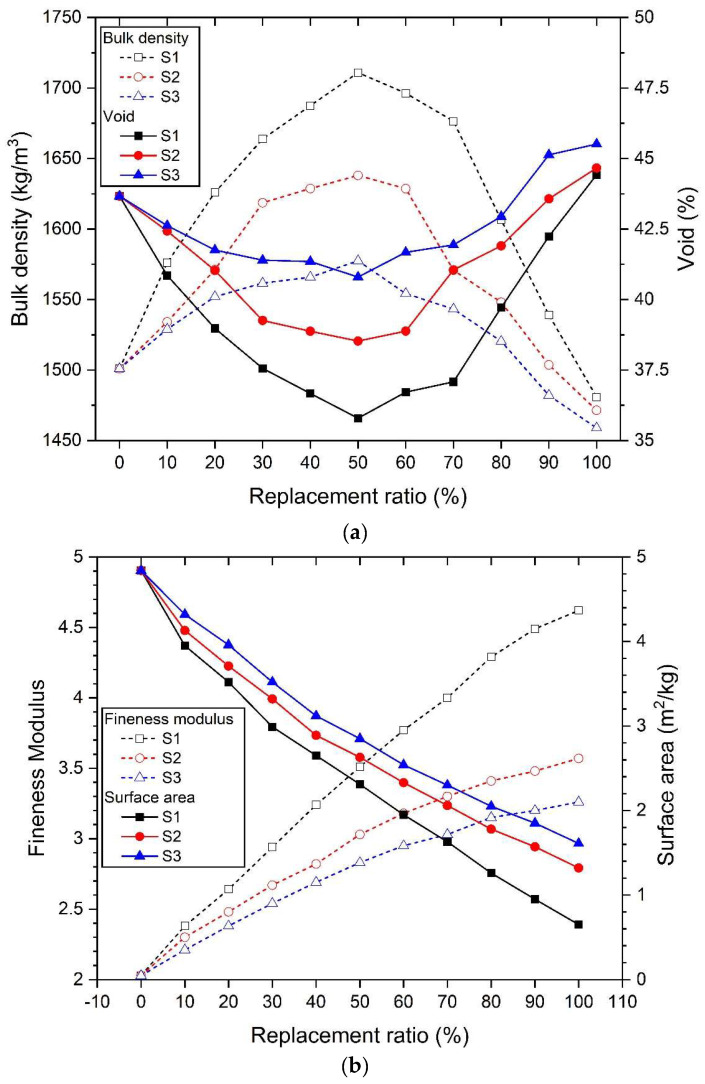
Characteristics of mixed fine aggregate: (**a**) bulk density and void, (**b**) fineness modulus and surface area.

Figure 3 shows the properties of mixed aggregate mixed with river sand and three types of silica sand of different sizes. Figure 3a shows both bulk density and void measurements. All three types of silica sand showed the highest bulk density and the smallest void value at a substitution rate of 50%. In other words, there was an inverse correlation between bulk density and void. If the bulk density is large and the void is small, the voids between the aggregates are small. This allows for the interlocking action of aggregates to support a large mechanical force in the entire mortar matrix. In particular, the mixture of S1 and river sand, which had the largest particle size among the three types, showed the highest bulk density and the lowest void value. Therefore, when mixing two types of fine aggregate, the optimal aggregate fraction is judged to be advantageous in forming a relatively more dense mortar matrix when the diameter ratio (*dr*) of the two particles is large. 

Figure 3b simultaneously shows the relationship between fineness modulus and surface area according to the replacement ratio of river sand and silica sand. Fineness modulus and surface area show conflicting relationships. Regardless of the size of the silica sand, as the replacement rate of silica sand increased, the surface area decreased and the fineness modulus increased. This is the result of the replacement of silica sand, which has larger particle sizes than river sand. In particular, S1 silica sand, which had the largest particle size, showed the smallest surface area and fineness modulus compared to the mixed fine aggregate that replaced the remaining S2 and S3 silica sands.

### 3.2. Workability (Flow Values)

**Figure 4:**
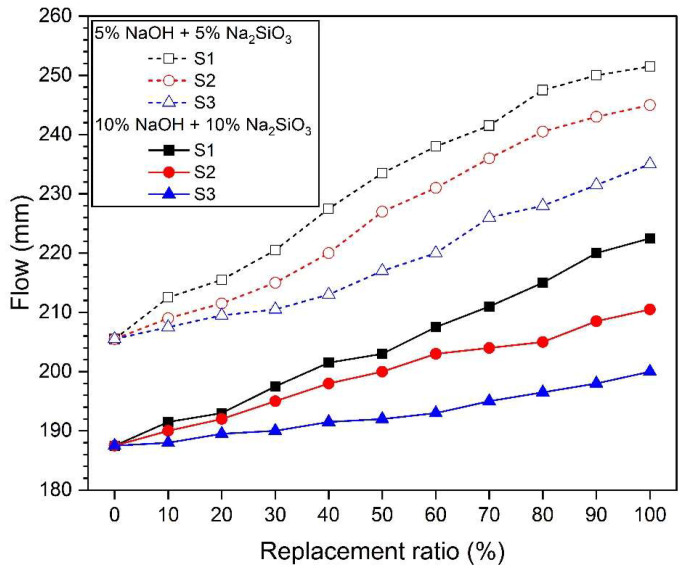
Flow values.

Figure 4 shows the flow values according to the replacement ratio of silica sands of different sizes. The flow value of the 10% concentration samples was lower than that of the 5% concentration activator mortar samples. This is because the higher the concentration of the activator, the more the hydration reaction of the slag is promoted, and the fluidity decreases due to the rapid formation of hydration reactants and coagulation. Also, regardless of the concentration of the activator, the larger the silica sand particles, the higher the flow value. In other words, in the order of S1 > S2 > S3, the larger the particle size of the silica sand, the larger the flow value shown at the same substitution ratio.

In general, the smaller the specific surface area of the fine aggregate, the higher the mortar flow [94,95]. As analyzed in Figure 3b, substitution of silica sand reduces the surface area of the mixed aggregate. In particular, the larger the particle size of silica sand replaced, the greater the effect of reducing the surface area of the mixed aggregate. Therefore, the samples replacing river sand with S1 have the smallest surface area, which shows the largest flow value, as shown in Figure 4. The increase in the fineness modulus of the fine aggregate shows a tendency consistent with previous research results showing that the workability of concrete increases due to a decrease in water demand due to a decrease in the total surface area [96,97,98]. In other words, the S1 substitution samples that show the lowest fineness modulus in Figure 3b show the highest flow value.

### 3.3. Compressive Strength

**Figure 5:**
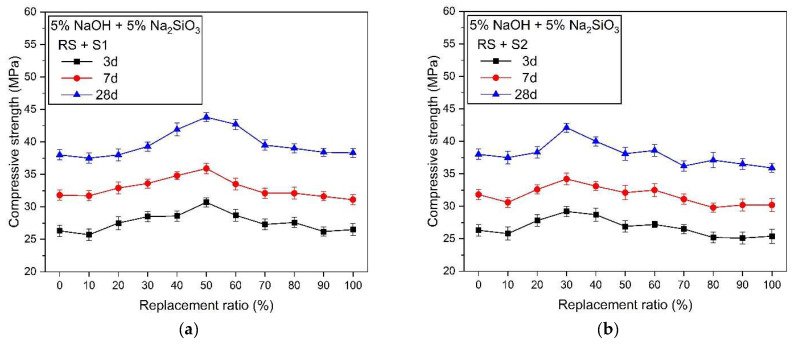

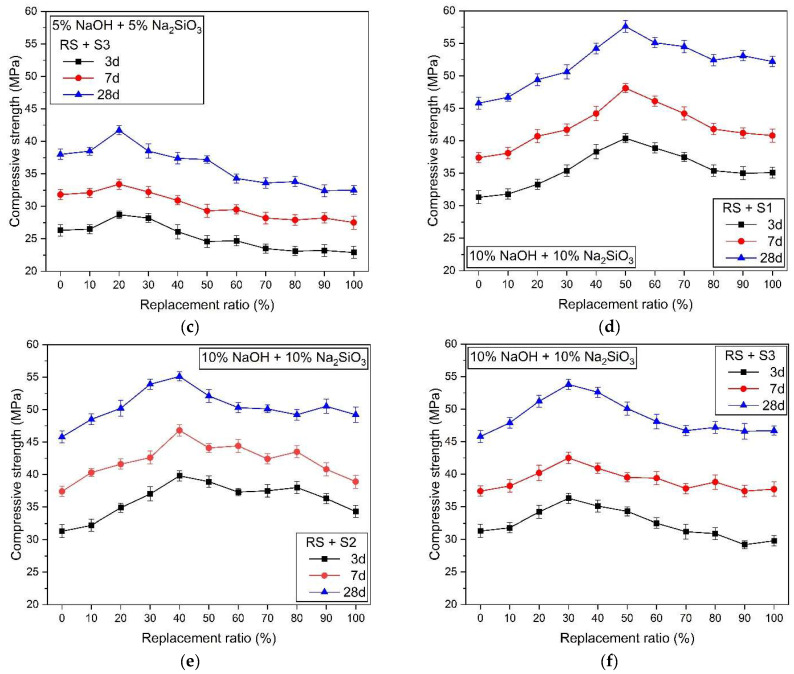
Compressive strength according to replacement ratio of river sand and silica sand, (**a**) 5% NaOH + 5% Na_2_SiO_3_, RS + S1, (**b**) 5% NaOH + 5% Na_2_SiO_3_, RS + S2, (**c**) 5% NaOH + 5% Na_2_SiO_3_, RS + S3, (**d**) 10% NaOH + 10% Na_2_SiO_3_, RS + S1, (**e**) 10% NaOH + 10% Na_2_SiO_3_, RS + S2, (**f**) 10% NaOH + 10% Na_2_SiO_3_, RS + S3.

Compressive strength characteristics according to the size and replacement ratio of silica sand are shown in Figure 5. Table 4 summarizes the highest strength values and substitution. Samples using a 5% concentration of the activator (Figure 5a–c) had lower compressive strength values at all measurement days compared to samples with 10% concentration (Figure 5d–f), regardless of the type and replacement rate of silica sand. This is because the lower the concentration of the alkaline activator in AASC, the slower the slag activation reaction, which prolongs the setting time and reduces the production of hydration reactants [15,17,99,100].

The change in strength varied depending on the size of the silica sand. Samples in which S1 silica sand was substituted for river sand showed a gradual increase in strength up to a 60% substitution rate, which then decreased as the substitution rate of silica sand increased (Figure 5a). That is, for the RS + S1 sample, the highest compressive strength value was measured at a 60% replacement rate on measurement days 3, 7, and 28. RS + S2 showed the highest substitution rate at 30% (Figure 5b) and RS + S3 at 20% (Figure 5c). As the size of the silica sand decreased in the order of S1 > S2 > S3, the substitution rate at which the highest strength value was found decreased in the order of 60% > 30% > 20%.

The change in the compressive strength of samples using a 10% concentration of alkali activator was different from that of the 5% concentration. The highest strength value was found at 50% replacement for RS + S1 samples (Figure 5d), a 40% replacement rate for RS + S2 samples (Figure 5e), and a 30% replacement rate for RS + S3 samples (Figure 5f). The intensity showed a gradual increase up to the highest value of the measured substitution rate and then decreased. 

The highest strength value and substitution rate can be clearly confirmed in Table 4. The substitution ratio, where the highest strength occurred in the sample using a 5% activator, gradually decreased to 30.7 > 29.2 > 28.7 MPa as the size of silica sand decreased from S1 to S2 to S3. For samples using a 10% concentration of alkali activator, the highest strength value gradually decreased in the order of 57.6 > 55.1 > 54.8 MPa as the size of the silica sand particles decreased from S1 to S2 to S3. It is believed that the change in the properties of the fine aggregate has a greater effect on the change in the strength value of AASC as the concentration of the alkali activator increases.

All three types of mixed aggregates substituted with silica sand of different sizes showed the highest bulk density and the lowest void at a 50% replacement ratio. The lowest void resulted in an insufficient amount of paste, forming pores in the ITZ or paste matrix and resulting in a decrease in mechanical performance [64,101,102]. However, the larger the diameter ratio (*dr*)—the larger the size of the silica sand—the greater the effect of the skeletal structure of the mixed aggregate on the mechanical properties compared to the effect of the lack of paste. Therefore, the highest intensity of RS +S1 occurs at a substitution ratio of 50%, but the highest intensity of RS + S2 and RS +S3 occurs at a substitution ratio of less than 50%.

Considering the difference in the silica sand replacement ratio where the highest strength occurred in the compressive strength results, it was confirmed that there is a specific value or range of substitution ratios of RS and silica sand where the structural stability of the AASC mortar is the highest. And, it was confirmed that this range or value changes depending on the size of the silica sand to be replaced. As a result, this means that predicting the mechanical performance of the actually manufactured AASC mortar based only on the physical properties of the mixed aggregate (fineness modulus, void, bulk density, surface area) may be inaccurate.

In general, the use of fine aggregates with large particle sizes also tends to promote the creation of voids in the AASC, resulting in a smaller effective area to resist compressive forces [103]. This results in a decrease in the mechanical performance of the AASC. However, when silica sand with a large particle size is mixed with river sand, an optimized filling state is formed according to the filling of the pores of the particles, thereby increasing the bulk density and lowering voids. The mixing effect of these aggregates forms a denser framework inside the AASC, thus improving mechanical performance [84]. As shown in Figure 3, the physical properties of mixed aggregate, which is a mixture of river sand and silica sand of three different sizes, show the highest bulk density and lowest void at a 50% replacement ratio. However, the highest actual strength was measured at substitution rates below 50%, except for S1, at 5% and 10% activator concentrations. This can be inferred from the following causes. (i) The mechanical properties of the mixed aggregate in Figure 3 are measured only for the aggregate. In other words, when the binder and water are mixed together with a viscous paste, the skeletal structure or arrangement of the aggregate may change. As a result, this may cause changes in the uniform distribution of the aggregate and the skeletal structure due to the viscosity of the paste. This requires considering the difference between the assumption that the aggregate will be homogeneously distributed inside the AASC mortar and the actual test specimen [103,104]. (ii) The adhesion between the aggregate and the paste. This affects the interfacial transition zone (ITZ) of the aggregate and the paste [101,104,105,106]. Adhesion is affected by the surface roughness, shape, and size of the aggregate. This must take into account the difference due to the idealization that assumes that river sand and silica sand are spherical. The two causes above are difficult to clearly measure and observe. Nevertheless, the compressive strength results confirm that there is a certain range or specific substitution ratio in which the mechanical performance of the mixed aggregate is the highest.

Table 5 shows the compressive strength values at each measurement age of samples mixed with river sand or silica sand alone. Here, RS is a mix using only 100% river sand without silica sand. And, S1, S2, and S3 are samples mixed only with silica sand with a 100% replacement ratio without river sand. Compared to RS, the 5% concentration alkali activator samples showed that the strength of all mortars using silica sand as the fine aggregate decreased compared to RS. The compressive strength decreased as the size of the silica sand decreased in the order of S > S2 > S3. However, for samples with a concentration of 10%, the strength of the mortar samples using silica sand was greater than that of the RS sample. The strength decreased as the size of the silica sand decreased in the order of S1 > S2 > S3, and the strength of the S3 samples showed similar values to RS. As the size of the silica sand particles became smaller, the compressive strength decreased. The strength of the mortar mixed with only S1, which has the largest particle size among the three types of silica sand used in the experiment, showed high compressive strength in AASC using 5% and 10% concentrations of alkali activator.

In Figure 3a, the bulk density of the mixed aggregate substituted with silica sand of three different sizes showed the maximum value at 50%, and the void value showed the minimum value at 50%. Therefore, it was predicted that the compressive strength of the mixed aggregate would be the highest at a replacement ratio of 50%, regardless of the type of silica sand. However, the actual measured compressive strength value was different than expected. Moreover, the substitution ratio at which the maximum strength value was measured varied depending on the size of the silica sand particles. This shows that in the case of mixed aggregate, there is a mixture sample in which the highest strength does not occur at a 50% replacement ratio, where the bulk density is the highest and the void is the lowest. This suggests that the factors affecting the mechanical performance of mixed aggregate may affect other characteristics in addition to the bulk density and void properties of the aggregate itself. In other words, there is a specific range of fineness modulus and surface area where the highest strength occurs, and this is believed to affect mechanical performance at the same time as bulk density and void.

**Figure 6:**
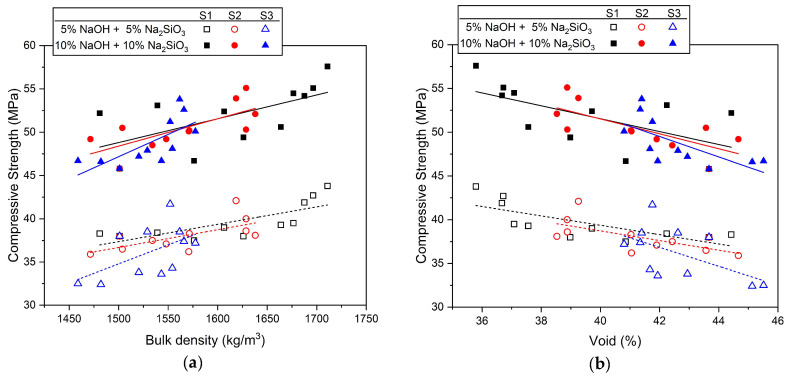

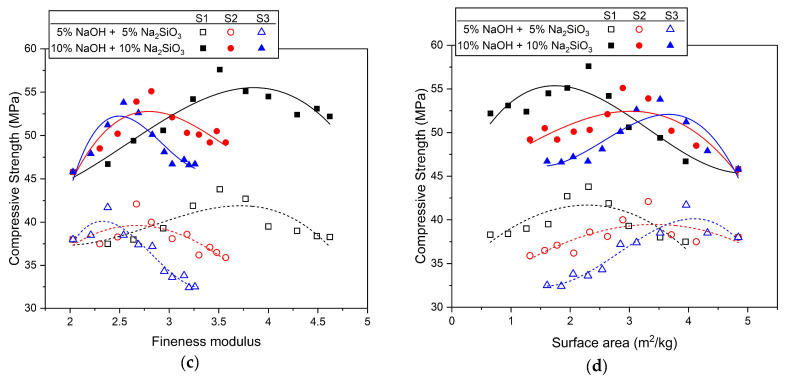
Correlation between the mechanical performance of mortar and the properties of mixed aggregate: (**a**) bulk density, (**b**) void, (**c**) fineness modulus, (**d**) surface area.

Figure 6 shows the correlation between the properties of mixed aggregate (fineness modulus, surface area, bulk density, and void) and compressive strength values. As shown in Figure 6a, the correlation between compressive strength and bulk density has a linear relationship. In other words, the larger the bulk density value, the greater the strength. The void and compressive strength in Figure 6b show a linear relationship, but as the void increases, the strength decreases. The mechanical properties of AASC mortar according to the bulk density and void of the mixed aggregate were found to have a larger distribution of strength values when the activator concentration was 10% than when the concentration was 5%.

It was observed that the fineness modulus in Figure 6c and the surface area in Figure 6d showed high strength within a specific value range. In Figure 6c, the highest compressive strength was observed for samples using a 5% concentration of activator when the fineness modulus values were 3.51 for S1, 2.67 for S2, and 2.38 for S3. Samples at a 10% concentration showed the highest values at 3.51 for S1, 2.82 for S2, and 2.54 for S3. In Figure 6d, the surface area values where the highest intensity of the 5% alkali activator concentration samples were measured were 2.31 m^2^/kg for S1, 3.32 m^2^/kg for S2, and 3.96 m^2^/kg for S3. For the samples with a 10% concentration, the values were 2.31 m^2^/kg for S1 and 2.89 m^2^/kg for S2, and S3 was 3.52 m^2^/kg. In other words, the highest strength of AASC using mixed aggregate was when the fineness modulus was 2.38–3.51 and the surface area was 2.31–3.96 m^2^/kg, which were values within a specific range.

As a result, it is judged that the fineness modulus and the surface area, as well as the void and the bulk density, should be considered important factors influencing the mechanical properties of AASC using mixed aggregate. In particular, it was confirmed that there is a specific value range for the fineness modulus and the surface area that demonstrates high strength.

### 3.4. Drying Shrinkage

**Figure 7:**
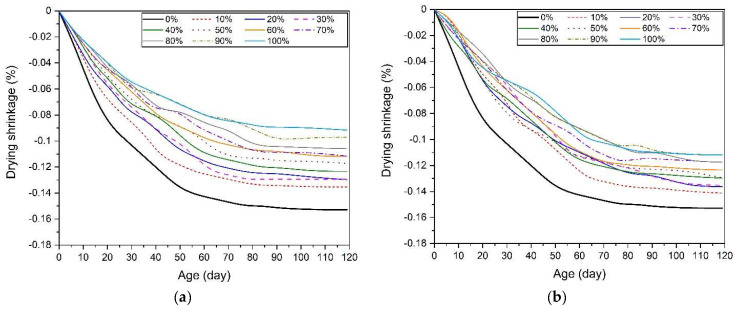

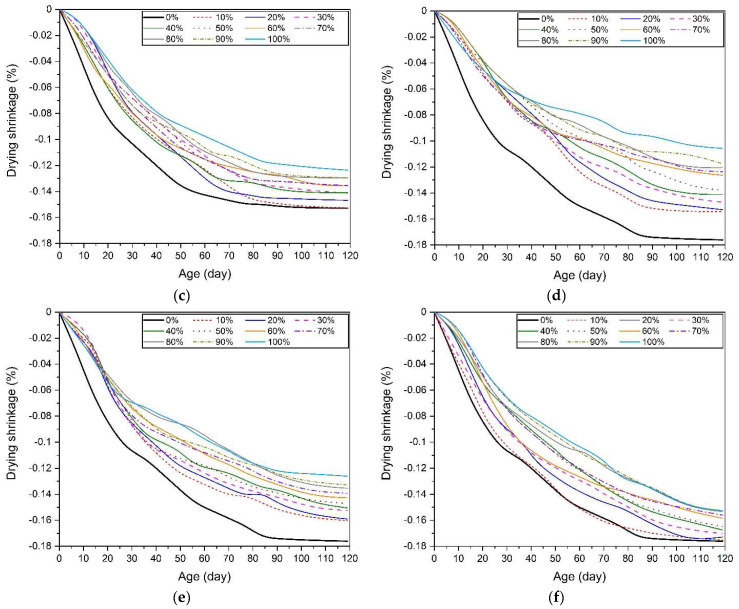
Drying shrinkage of AASC mortar using mixed aggregate, (**a**) 5% NaOH + 5% Na_2_SiO_3_, RS + S1, (**b**) 5% NaOH + 5% Na_2_SiO_3_, RS + S2, (**c**) 5% NaOH + 5% Na_2_SiO_3_, RS + S3, (**d**) 10% NaOH + 10% Na_2_SiO_3_, RS + S1, (**e**) 10% NaOH + 10% Na_2_SiO_3_, RS + S2, (**f**) 10% NaOH + 10% Na_2_SiO_3_, RS + S3.

Figure 7 shows the drying shrinkage measurement results of AASC mortar mixed with river sand and silica sand of three different sizes. Regardless of the concentration of the alkali activator and the size of the silica sand, the drying shrinkage rate decreased when river sand was replaced with silica sand. The drying shrinkage reduction rate showed a greater shrinkage reduction effect as the replacement ratio of silica sand increased. Therefore, the drying shrinkage rate of AASC mortar using 100% silica sand (without river sand) as a fine aggregate showed the lowest value.

For samples using a 5% concentration of activator, the change in drying shrinkage became insignificant after about 70 days (Figure 7a–c). However, for samples at 10% concentration, the change in shrinkage rate became insignificant after about 90 days (Figure 7d–f). Additionally, the alkali activator sample with a 10% concentration showed a greater shrinkage rate than the sample with a 5% concentration of alkaline activator [22,45,46,47,100,107].

Table 6 summarizes the drying shrinkage rates measured at 120 days. The drying shrinkage rate of AASC mortar using only river sand at 120 days was −0.1528% for the 5% concentration sample and −0.1760% for the 10% concentration sample. The larger the size of the silica sand substituted for river sand, the larger and more clearly the effect of reducing drying shrinkage. In other words, samples substituted with river sand and S1 showed the greatest reduction in water downturn compared to samples substituted with the remaining S2 and S3 silica sands. And, the sample using 100% S1 showed the smallest drying shrinkage rate among all samples. Samples substituted with S3, the smallest size among the three types of silica sand, had a lower shrinkage reduction effect compared to samples substituted with S1 and S2. These shrinkage characteristics were consistent with reports from previous studies that showed relatively smaller drying shrinkage as the size of the aggregate increased under the same binder content [84]. According to a study by Karagüler and Yatağan [108], the use of aggregates with large particle sizes showed the effect of suppressing shrinkage by reducing the volume change of hydration reactants (typically C(A)SH gel) in the drying environment of AASC. This shows that using mixed aggregates of various aggregate sizes can affect the drying shrinkage inhibition effect of AASC.

**Figure 8:**
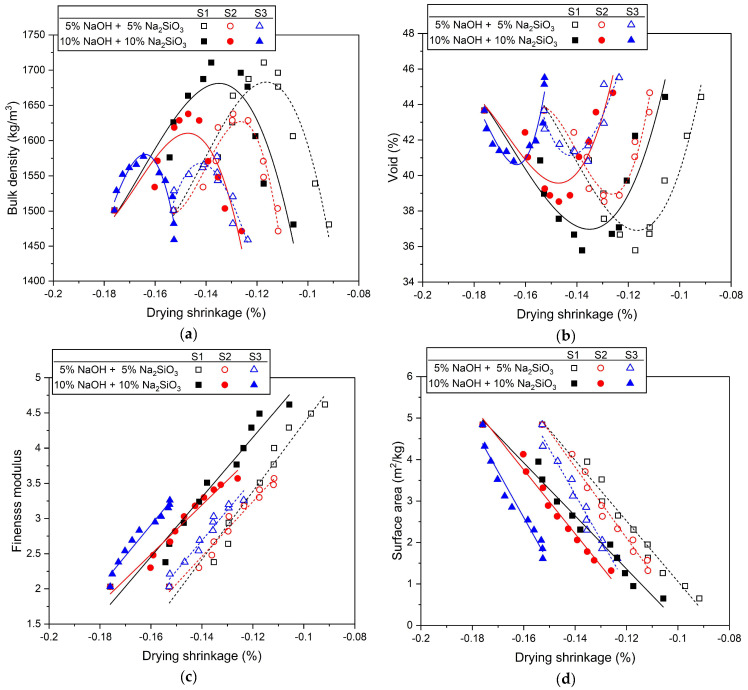
Correlation between drying shrinkage and properties of mixed aggregates: (**a**) bulk density, (**b**) void, (**c**) fineness modulus, (**d**) surface area.

Figure 8 shows the correlation between drying shrinkage and the properties of mixed aggregate. Figure 8a shows the relationship between bulk density and shrinkage rate. The highest bulk density value occurs at a 50% replacement ratio for all three types of silica sand. Drying shrinkage tends to gradually decrease when the silica sand replacement ratio exceeds 50%. Also, in Figure 8b, the void also has the lowest value at a substitution rate of 50%, but a water reduction effect appears at a substitution rate exceeding 50%. As a result, at the replacement ratio where the skeletal structure of the mixed aggregate is most optimized, in other words, the substitution ratio where the bulk density is the highest and the void is the smallest, it does not show the smallest drying shrinkage. The fineness modulus and shrinkage rate in Figure 8c showed a linear, proportional relationship. In other words, as the fineness modulus increased, the drying shrinkage decreased. As the substitution rate of silica sand increases, the average particle size of the mixed aggregate increases, which increases the fineness modulus. Figure 8d shows the relationship between the surface area and the drying shrinkage rate, and it can be confirmed that they show an inverse linear relationship. In other words, as the surface area of the mixed aggregate decreased, the drying shrinkage decreased. The decrease in the surface area of the mixed aggregate is because the average size of the aggregate particles increased with the replacement of silica sand.

Through the results in Figure 8c,d, it was clearly confirmed that the larger the particle size of the mixed aggregate, the lower the drying shrinkage. The shrinkage reduction effect of AASC using large-sized aggregates is already known through the results of previously published studies [84,109], and the results shown in this study are consistent with the trends of these previous studies.

In order to analyze the effect on the drying shrinkage of AASC, this study examined the effect of mixed aggregate mixed with river sand and silica sand of three different sizes. However, the pore structure and distribution of AASC paste, which were not considered in this study, and the investigation and influence of high capillary pore pressure, reconstructed structure, and surface-free energy according to pore characteristics are beyond the scope of this study [24,35,36]. Nevertheless, despite the limited conditions and experimental considerations that only focused on mixed aggregate, it was confirmed that mixed aggregates of various sizes were effective in controlling the drying shrinkage of AASC.

### 3.5. Total Porosity

**Figure 9:**
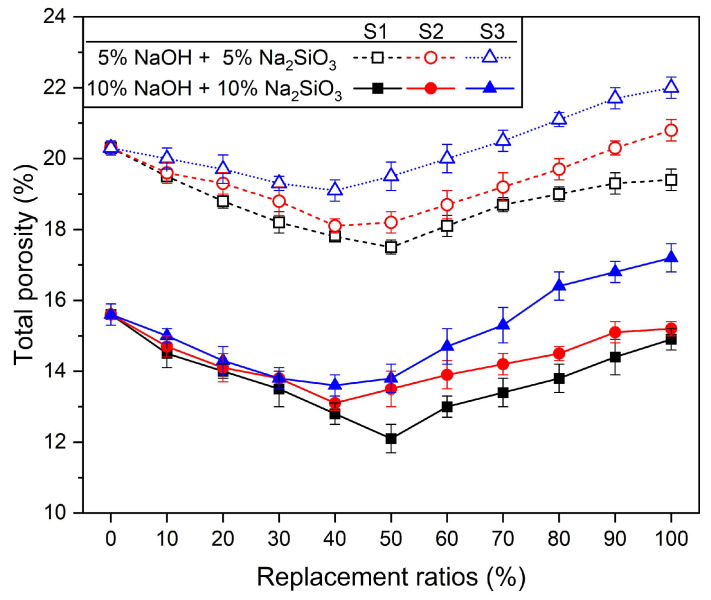
Total porosity according to the type and replacement rate of silica sand.

Figure 9 shows the change in total porosity according to the three types of silica sand and silica sand replacement ratios. In Figure 9, regardless of the type and replacement rate of silica sand, 10% NaOH + 10% Na_2_SiO_3_ specimens show lower porosity than the 5% NaOH + 5% Na_2_SiO_3_ specimen. This means that when the concentration of the activator is high in AASC, the hydration reaction of the slag is promoted, the amount of hydration reactant formation is improved, and a dense matrix is formed [17,99,100].

Compared to the 100% RS sample using 100% river sand, the total porosity increased in all mixes in which the two types of silica sand were substituted. This was observed simultaneously at 5% and 10% alkaline activator concentrations. In particular, as the size of silica sand decreased, the total porosity increased. In other words, the total porosity of samples where silica sand was replaced in the order S3 > S2 > S1 increased. In other words, as the particle size of the substituted silica sand decreased, the total porosity increased. As shown in the characteristics of the mixed aggregate and compressive strength characteristics in Figure 3 and Figure 5, it was expected that the total porosity would show characteristics depending on the size and replacement rate of silica sand. 

The minimum total porosity at the concentration and substitution ratio of the alkaline activator was similar to the substitution ratio at which the highest compressive strength occurred, but it did not match. As shown in the characteristics of the mixed aggregate in Figure 3, the smallest void and the largest bulk density were observed when the silica sand replacement ratio was 50%. Therefore, it was expected that the smallest total porosity would be measured at a 50% substitution rate, regardless of the type of silica sand and the concentration of the alkali activator. At 5% NaOH + 5% Na_2_SiO_3_, samples with S1 substitution showed the lowest total porosity value of 17.5% at a 50% substitution ratio. Samples with S2 substitution showed the lowest total porosity value of 18.1% at a 40% substitution rate, and samples with S3 substitution showed the lowest total porosity value of 19.1% at a 40% substitution rate. As shown in Table 4, the highest compressive strength occurred at 50%, 30%, and 20%, which is different from the replacement rate at which the minimum total porosity occurred. At 10% NaOH + 10% Na_2_SiO_3_, the mixed samples of S1 and RS had the lowest porosity values of 12.1% at a 50% replacement rate, S2 at 13.1% at a 40% replacement rate, and S3 at 13.6% at a 40% replacement rate. In Table 4, the highest compressive strengths of the S1 + RS, S2 + RS, and S3 + RS samples occurred at 50%, 40%, and 30% replacement ratios. Regardless of the concentration of the activator, there was a slight difference in the substitution rate at which the highest compressive strength occurred and the substitution rate at which the minimum total porosity occurred. This is believed to be due to unpredictable problems, such as the distribution and homogeneity of the aggregate due to the viscous paste being mixed with the mixed aggregate in AASM [103,104].

Another cause that can be considered for the irregular and inconsistent complex changes in the pore structure of AASM using mixed aggregate of RS and silica sand is the influence of the interfacial transition zone (ITZ), which is the interface between the aggregate and paste [101,104,105,106]. The total porosity measurement results shown in this study are not sufficient to explain the mechanical properties and drying shrinkage rate of AASM mortar according to the three types of silica sand with different particle sizes and substitution ratios. First, in terms of mechanical properties, the total porosity in the specimens where the highest strength occurred was greater than that of 100% river sand (RS) specimens but smaller than that of 100% silica sand. According to the size of the total porosity, the compressive strength of the 100% RS specimen should have been the highest, but this was not the case. In other words, the physical properties of mixed aggregate do not have a direct correlation to the mechanical performance of AASC mortar. This is because, as previously mentioned, the skeletal structure and dispersion of mixed aggregate may change in irregular and unexpected directions due to mixing of binder and water, and this is reflected in the mechanical properties [104]. Nevertheless, a significant correlation was observed between the physical properties of the mixed aggregate and the drying shrinkage in samples replaced with 100% silica sand. The 100% silica sand replacement samples showed the highest total porosity, void, and fineness modulus and the lowest bulk density and surface area. Under the same mixing conditions, as the diameter of the aggregate increases, the diameter of the pores increases, which reduces the meniscus of water compared to the capillary pores and thus reduces drying shrinkage [33,84,101]. When aggregates with large particle sizes are used, the pore number of the AASC increases, which is stored in the internal pores of the AASC when the same amount of water evaporates. If the size or quantity of the pores is larger, the meniscus radius of water increases, the capillary pore pressure decreases, and the contraction pressure decreases [84]. Therefore, the drying shrinkage of specimens mixed with S1, which has the largest size of silica sand, was found to be the smallest.

From reports of previous studies that studied the influence of many aggregates, the influence of the interfacial transition zone (ITZ), the boundary between the aggregate and the paste, was not clearly considered in this study. To date, there are many factors that affect the ITZ, and the characteristics of the ITZ are known to affect the mechanical performance of mortar or concrete [104,105,110,111]. However, clear measurement methods, evaluation factors, influencing factors, and mechanisms for ITZ have not yet been identified [104,105,112]. Previous studies have shown that the particle size and fraction of aggregate affect the properties of ITZ, which in turn affect the porosity, strength, and durability of mortar or concrete [110,112]. ITZ also partially contributes to the increase in porosity of specimens [106]. However, in order to examine the impact on ITZ, which is the interface between the aggregate and the paste, analysis of countless ITZs is required, which causes problems of time and cost [110]. Because the measurement of total porosity includes both aggregate particles and paste, it naturally also includes ITZ. Because porosity is measured in the paste and the ITZ, the results vary significantly depending on the aggregate fraction of the sample used to measure the total porosity [101]. However, what is clear is that as the size of silica sand particles decreases, the total porosity increases (Figure 9). In other words, the total porosity increases in the order S3 > S2 > S1, which means that with finer aggregate particles, the specific surface area increases and the amount of ITZ increases, which will have a certain effect on the increase in total porosity [104]. From some other perspectives, some research results suggest that the increase in porosity of specimens is due to the increase in ITZ because the porosity of ITZ is larger compared to paste [84]. Therefore, as the size of silica sand decreases, the surface area increases, so it can be inferred that ITZ pores were partially involved in the increase in total porosity. This inference can be explained as one of the reasons why the total porosity of specimens containing S3 or using S3 alone showed a large value compared to other specimens.

However, the relationship between compressive strength and drying shrinkage is not clear. As mentioned in this text, this is influenced by various factors. The viscosity of the paste may cause changes in the homogeneous distribution of the aggregate and skeletal structure during mixing [103,104]. In other words, the actual distribution and design of aggregate in the test specimen may differ from the ideal distribution. Additionally, due to differences in adhesion between the aggregate and the paste, adhesion is affected by the surface roughness, shape, and size of the aggregate [104,105,106]. And, changes in the pore structure also affect the adhesion between the aggregate and the paste. Microstructurally, there is also a complex effect due to the interfacial transition zone (ITZ) of the aggregate and the paste [101,102,106,110]. The purpose of this study was to test the effect of reducing the drying shrinkage of AASC and its characteristics based on the characteristics of the aggregate. Measurement of the homogeneity of ITZ and the aggregate distribution cannot be determined from the experimental elements and measurement range of this study, and reports from previous studies were referenced. This is planned to be addressed in more detail in follow-up research.

## 4. Conclusions

The effect on the strength and drying shrinkage of alkali-activated slag cement mortar using mixed aggregates replacing river sand and silica sand in three sizes was investigated.

(1) The physical properties of the mixed aggregate of three types of river sand and silica sand were not proportional to the mechanical properties of AASC mortar. This means that the skeleton and distribution of the aggregate are affected by the mix of the mixed aggregate and paste. However, the strength improvement effect was confirmed when the fineness modulus of the mixed aggregate was 2.25 to 3.75 and the surface area was 2.25 to 4.25 m^2^/kg.
(2) When river sand and three types of silica sand were used alone, the larger the particle size of the aggregate, the greater the effect of reducing drying shrinkage (S1 > S2 > S3 > RS). When part of the river sand was replaced with silica sand, the larger the size of the silica sand particles, the greater the effect of reducing drying shrinkage (RS + S1 > RS + S2 > RS + S3). As a result, it is believed that replacing 50% or more of silica sand with a particle size larger than that of river sand could be an effective method for reducing drying shrinkage.
(3) In the case of mixed aggregate, there was no clear pore structure tendency because it had a complex effect on the properties of the paste and the aggregate. As a result, the unclear pore structure makes it difficult to clearly explain the mechanisms and mutual influences of mechanical performance and shrinkage reduction. However, considering the mechanical properties and drying shrinkage, when mixing river sand and silica sand of different sizes, the larger the particle size ratio (*dr*) of the two fine aggregates, the more effective it is.

The results of this study suggested the possibility of reducing the drying shrinkage of AASC by using only mixed aggregate without changing the hydration reactant in a different way from previous studies (use of shrinkage-reducing agents or additional admixtures). It is expected that it could be used as a more effective shrinkage reduction method for AASC when used in combination with other existing shrinkage-reduction methods. It is believed that the results of this study can be used as basic data for designing the quality and mix of aggregates to reduce drying shrinkage in the manufacturing of AAS concrete using both fine and coarse aggregates.

## Figures and Tables

**Table 1:** Chemical component properties in slag.

	Chemical Components (%)						
	SiO_2_	Al_2_O	Fe_2_O	MgO	CaO	K_2_O	SO_3_
Slag	34.87	8.52	0.79	3.81	46.95	0.43	3.74

**Table 2:** The properties of river sand and silica sands.

	River Sand (RS)	Silica Sand1 (S1)	Silica Sand2 (S2)	Silica Sand3 (S3)
Aggregate size range (mm)	0.20–0.80	2.50–5.00	1.60–2.50	1.12–1.60
Mean diameter (*d_m_*, mm)	0.45	3.46	1.69	1.39
Fineness modulus (FM)	2.03	4.62	3.57	3.26
Absorption (%)	1.01	0.42	0.42	0.42
Specific surface area (m^2^/kg)	4.84	0.65	1.32	1.61
Density (g/cm^3^)	2.75	2.67	2.67	2.67
Diameter ratio (*dr*)	–	7.68	3.75	3.02

**Table 3:** Mixing ratios (g).

Binder (Slag)	Alkali-Solution	River Sand	Silica Sand	Replacement Ratio (%)
		2000	0	0
		1800	200	10
		1600	400	20
		1400	600	30
		1200	800	40
1000	500	1000	1000	50
		800	1200	60
		600	1400	70
		400	1600	80
		200	1800	90
		0	2000	100

**Table 4:** Highest strength value and substitution rate.

	Replacement Ratios (%)	Highest Strength (MPa)		
		3d	7d	28d
5% NaOH + 5% Na_2_SiO_3_				
RS + S1	50	30.7	35.9	43.8
RS + S2	30	29.2	34.2	42.1
RS + S3	20	28.7	33.4	41.6
10% NaOH + 10% Na_2_SiO_3_				
RS + S1	50	40.4	48.1	57.6
RS + S2	40	39.8	46.8	55.1
RS + S3	30	36.3	42.5	53.8

**Table 5:** Strength of samples mixed with river sand or silica sand alone (MPa).

	Age (Day)	RS (Without Silica Sand)	S1	S2	S3
			(Without River Sand)		
5% NaOH + Na_2_SiO_3_	3	26.3	26.5	25.4	22.9
	7	31.8	31.1	30.2	27.5
	28	38.0	38.3	35.9	32.5
10% NaOH + Na_2_SiO_3_	3	31.3	35.1	34.3	29.8
	7	37.4	40.8	38.9	37.7
	28	45.8	52.2	49.2	46.7

**Table 6:** Summary of final drying shrinkage measured at 120 days for AASC mortar using mixed aggregate.

	5% NaOH + 5% Na_2_SiO_3_			10% NaOH + 10% Na_2_SiO_3_		
Replacement Ratio (%)	RS + S1	RS + S2	RS + S3	RS + S1	RS + S2	RS + S3
0	−0.1528			−0.1760		
10	−0.1353	−0.1412	−0.1526	−0.1543	−0.1602	−0.1752
20	−0.1297	−0.1361	−0.1468	−0.1528	−0.1591	−0.1728
30	−0.1295	−0.1353	−0.1413	−0.1471	−0.1525	−0.1702
40	−0.1233	−0.1297	−0.1409	−0.1411	−0.1505	−0.1674
50	−0.1173	−0.1294	−0.1357	−0.1380	−0.1470	−0.1646
60	−0.1118	−0.1235	−0.1356	−0.1264	−0.1427	−0.1584
70	−0.1117	−0.1175	−0.1354	−0.1237	−0.1392	−0.1560
80	−0.1059	−0.1174	−0.1295	−0.1206	−0.1353	−0.1532
90	−0.0972	−0.1119	−0.1295	−0.1174	−0.1326	−0.1527
100	−0.0917	−0.1117	−0.1236	−0.1057	−0.1260	−0.1526

## Data Availability

The original contributions presented in the study are included in the article, further inquiries can be directed to the corresponding author.

## References

[1] Worrell E., Price L., Martin N., Hendriks C., Meida L.O. (2001). Carbon dioxide emission from the global cement industry. Annu. Rev. Energy Environ..

[2] Hendriks C.A., Worrell E., De Jager D. Emission reduction of greenhouse gases from the cement industry. Proceedings of the 7th International Conference on Greenhouse Gas Control Technologies.

[3] Ding Y., Dai J.G., Shi C.J. (2016). Mechanical properties of alkali-activated concrete: A state-of-the-art review. Constr. Build. Mater..

[4] Gartner E. (2004). Industrially interesting approaches to “low-CO_2_” cements. Cem. Concr. Res..

[5] Rashad A.M. (2013). A comprehensive overview about the influence of different additives on the properties of alkali-activated slag—A guide for Civil Engineer. Constr. Build. Mater..

[6] Schneider M. (2019). The cement industry on the way to a low-carbon future. Cem. Concr. Res..

[7] Shi C., Roy D., Krivenko P. (2006). Alkali-Activated Cements and Concretes.

[8] Provis J.L., Palomo A., Shi C. (2015). Advances in understanding alkali-activated materials. Cement Concr. Res..

[9] Habert G., Miller S.A., John V.M., Provis J.L., Favier A., Horvath A., Scrivener K.L. (2020). Environmental impacts and decarbonization strategies in the cement and concrete industries. Nat. Rev. Earth Environ..

[10] Provis J.L. (2018). Alkali-activated materials. Cement Concr. Res..

[11] Duxson P., Fernández-Jiménez A., Provis J.L., Lukey G.C., Palomo A., Deventer J. (2007). Geopolymer technology: The current state of the art. J. Mater. Sci..

[12] Provis J.L., Van Deventer J.S.J. (2009). Geopolymers: Structures, Processing, Properties and Industrial Applications.

[13] Shi C., Stegemann J.A. (2000). Acid corrosion resistance of different cementing materials. Cem. Concr. Res..

[14] Zhang J., Shi C., Zhang Z., Ou Z. (2017). Durability of alkali-activated materials in aggressive environments: A review on recent studies. Constr. Build. Mater..

[15] He J., Li M., Bai W., Sang G. (2024). Effect of slaked lime on the properties of sodium sulfate-activated alkali-activated slag cement. Materials.

[16] Abdullah M.N., Mustapha F., Yusof N.I., Khan T., Sebaey T.A. (2024). Thermal Properties and Drying Shrinkage Performance of Palm Kernel Shell Ash and Rice Husk Ash-Based Geopolymer Concrete. Materials.

[17] Zamanabadi S.N., Zareei S.A., Shoaei P., Ameri F. (2019). Ambient-cured alkali-activated slag paste incorporating micro-silica as repair material: Effects of alkali activator solution on physical and mechanical properties. Constr. Build. Mater..

[18] Ye H., Radlińska A. (2017). Carbonation-induced volume change in alkali-activated slag. Constr. Build. Mater..

[19] Puertas F., Palacios M., Vázquez T. (2006). Carbonation process of alkali-activated slag mortars. J. Mater. Sci..

[20] Bakharev T., Sanjayan J., Cheng Y.-B. (2001). Resistance of alkali-activated slag concrete to carbonation. Cem. Concr. Res..

[21] Wang S.-D., Pu X.-C., Scrivener K.L., Pratt P.L. (1995). Alkali-activated slag cement and concrete: A review of properties and problems. Adv. Cem. Res..

[22] Atis C.D., Bilim C., Çelik Ö., Karahan O. (2009). Influence of activator on the strength and drying shrinkage of alkali-activated slag mortar. Constr. Build. Mater..

[23] Bakharev T., Sanjayan J.G., Cheng Y.B. (1999). Alkali activation of Australian slag cements. Cem. Concr. Res..

[24] Kumarappa D.B., Peethamparan S., Ngami M. (2018). Autogenous shrinkage of alkali activated slag mortars: Basic mechanisms and mitigation methods. Cem. Concr. Res..

[25] Humad A.M., Kothari A., Provis J.L., Cwirzen A. (2019). The effect of blast furnace slag/fly ash ratio on setting, strength, and shrinkage of alkali-activated pastes and concretes. Front. Mater..

[26] Zhang B., Zhu H., Cheng Y., Huseien G.F., Shah K.W. (2022). Shrinkage mechanisms and shrinkage-mitigating strategies of alkali-activated slag composites: A critical review. Constr. Build. Mater..

[27] Mastali M., Kinnunen P., Dalvand A., Mohammadi Firouz R., Illikainen M. (2018). Drying shrinkage in alkali-activated binders—A critical review. Constr. Build. Mater..

[28] Li Z., Liu J., Ye G. (2019). Drying shrinkage of alkali-activated slag and fly ash concrete; a comparative study with ordinary Portland cement concrete. Heron.

[29] Wu H. (2023). Improving Freeze-Thaw Resistance of Alkali-Activated Slag by Admixtures. Master’s Thesis.

[30] Bernal S.A., de Gutierrez R.M., Provis J.L., Rose V. (2010). Effect of silicate modulus and metakaolin incorporation on the carbonation of alkali silicate-activated slags. Cem. Concr. Res..

[31] Hojati M., Radlińska A. (2017). Shrinkage and strength development of alkali-activated fly ash-slag binary cements. Constr. Build. Mater..

[32] Palacios M., Puertas F. (2007). Effect of shrinkage-reducing admixtures on the properties of alkali-activated slag mortars and pastes. Cem. Concr. Res..

[33] Collins F., Sanjayan J.G. (2000). Effect of pore size distribution on drying shrinking of alkali-activated slag concrete. Cem. Concr. Res..

[34] Zhang B., Zhu H., Feng P., Zhang P. (2022). A review on shrinkage-reducing methods and mechanisms of alkali-activated/geopolymer systems: Effects of chemical additives. J. Build. Eng..

[35] Li Z., Lu T., Liang X., Dong H., Ye G. (2020). Mechanisms of autogenous shrinkage of alkali-activated slag and fly ash pastes. Cem. Concr. Res..

[36] Hojati M., Rajabipour F., Radlińska A. (2019). Drying shrinkage of alkali-activated cements: Effect of humidity and curing temperature. Mater. Struct..

[37] Lura P., Jensen O.M., van Breugel K. (2003). Autogenous shrinkage in highperformance cement paste: An evaluation of basic mechanisms. Cem. Concr. Res..

[38] Altan E., Erdoǧan S.T. (2012). Alkali activation of a slag at ambient and elevated temperatures. Cem. Concr. Compos..

[39] Aydin S., Baradan B. (2012). Mechanical and microstructural properties of heat cured alkali-activated slag mortars. Mater. Des..

[40] Ye H., Cartwright C., Rajabipour F., Radlińska A. (2017). Understanding the drying shrinkage performance of alkali-activated slag mortars. Cem. Concr. Compos..

[41] Fang S., Lam E.S.S., Li B., Wu B. (2020). Effect of alkali contents, moduli and curing time on engineering properties of alkali activated slag. Constr. Build. Mater..

[42] Lahalle H., Benavent V., Trincal V., Wattez T., Bucher R., Cyr M. (2021). Robustness to water and temperature, and activation energies of metakaolin-based geopolymer and alkali-activated slag binders. Constr. Build. Mater..

[43] Bakharev T., Sanjayan J., Cheng Y.-B. (1999). Effect of elevated temperature curing on properties of alkali-activated slag concrete. Cem. Concr. Res..

[44] Douglas E., Bilodeau A., Malhotra V. (1992). Properties and durability of alkali-activated slag concrete. ACI Mater. J..

[45] Chen W., Li B., Wang J., Thom N. (2021). Effect of alkali-dosage and silicate modulus on autogenous shrinkage of alkali-activated slag cement paste. Cem. Concr. Res..

[46] Melo Neto A.A., Cincotto M.A., Repette W. (2008). Drying and autogeous shrinkage of pastes and mortars with acivated slag cement. Cem. Concr. Res..

[47] Das S.K., Shrivastava S. (2021). Influence of molarity and alkali mixture ratio on ambient temperature cured waste cement concrete based geopolymer mortar. Constr. Build. Mater..

[48] Collins F., Sanjayan J.G. (1999). Strength and shrinkage properties of alkali-activated slag concrete containing porous coarse aggregate. Cem. Concr. Res..

[49] Sakulich A., Bentz D. (2013). Mitigation of autogenous shrinkage in alkali activated slag mortars by internal curing. Mater. Struct..

[50] Ye H., Cartwright C., Rajabipour F., Radlińska A. Effect of drying rate on shrinkage of alkali-activated slag cements. Proceedings of the 4th International Conference on the Durability of Concrete Structure (ICDCS) Purdue University.

[51] Ma J., Dehn F. (2017). Shrinkage and creep behavior of an alkali-activated slag concrete. Struct. Concr..

[52] You N., Liu Y., Gu D., Ozbakkaloglu T., Pan J., Zhang Y. (2020). Rheology, shrinkage and pore structure of alkali-activated slag-fly ash mortar incorporating copper slag as fine aggregate. Constr. Build. Mater..

[53] Dueramae S., Tangchirapat W., Chindaprasirt P., Jaturapitakkul C., Sukontasukkul P. (2020). Autogenous and drying shrinkages of mortars and pore structure of pastes made with activated binder of calcium carbide residue and fly ash. Constr. Build. Mater..

[54] Liu J., Hu L., Tang L., Zhang E.Q., Ren J. (2020). Shrinkage behaviour, early hydration and hardened properties of sodium silicate activated slag incorporated with gypsum and cement. Constr. Build. Mater..

[55] Chen W., Brouwers H.J.H. (2012). Hydration of mineral shrinkage-compensating admixture for concrete: An experimental and numerical study. Constr. Build. Mater..

[56] Shi C. (1996). Strength, pore structure and permeability of alkali-activated slag mortars. Cem. Concr. Res..

[57] Yang L., Jia Z., Zhang Y., Dai J. (2015). Effects of nano-TiO_2_ on strength, shrinkage and microstructure of alkali-activated slag pastes. Cem. Concr. Compos..

[58] Ye H., Radlińska A. (2017). Shrinkage mitigation strategies in alkali-activated slag. Cem. Concr. Res..

[59] Lecomte I., Henrist C., Liegeois M., Maseri F., Rulmont A., Cloots R. (2006). (Micro)-structural comparison between geopolymers, alkali-activated slag cement and Portland cement. J. Eur. Ceram. Soc..

[60] Puertas F., Gil-Maroto A., Palacios M., Amat T. (2006). Alkali-activated slag mortars reinforced with ar glassfibre. Performance and properties. Mater. Constr..

[61] Puertas F., Amat T., Ferna A. (2003). Mechanical and durable behaviour of alkaline cement mortars reinforced with polypropylene fibres. Cement. Conc. Res..

[62] Aydın S., Baradan B. (2013). The effect of fiber properties on high performance alkali activated slag/silica fume mortars. Compos. Part B Eng..

[63] Almakhadmeh M., Soliman A.M. (2021). Effects of mixing water temperatures on properties of one-part alkali-activated slag paste. Constr. Build. Mater..

[64] Li Z., Nedeljković M., Chen B., Ye G. (2019). Mitigating the autogenous shrinkage of alkali-activated slag by metakaolin. Cem. Concr. Res..

[65] Rostami M., Behfarnia K. (2017). The effect of silica fume on durability of alkali activated slag concrete. Constr. Build. Mater..

[66] Deb P.S., Nath P., Sarker P.K. (2015). Drying shrinkage of slag blended fly ash geopolymer concrete cured at room temperature. Procedia Eng..

[67] Ma Y., Ye G. (2015). The shrinkage of alkali activated fly ash. Cem. Concr. Res..

[68] Yuan X., Chen W., Lu Z., Chen H. (2014). Shrinkage compensation of alkali-activated slag concrete and microstructural analysis. Constr. Build. Mater..

[69] Jia Z., Yang Y., Yang L., Zhang Y., Sun Z. (2018). Hydration products, internal relative humidity and drying shrinkage of alkali activated slag mortar with expansion agents. Constr. Build. Mater..

[70] Jin F., Al-Tabbaa A. (2015). Strength and drying shrinkage of slag paste activated by sodium carbonate and reactive MgO. Constr. Build. Mater..

[71] Abdel-Gawwad H.A., Mohammed M.S., Alomayri T. (2019). Single and dual effects of magnesia and alumina nano-particles on strength and drying shrinkage of alkali activated slag. Constr. Build. Mater..

[72] Shahrajabian F., Behfarnia K. (2018). The effects of nano particles on freeze and thaw resistance of alkali-activated slag concrete. Constr. Build. Mater..

[73] Liu B., Yang J., Li D., Xing F., Fang Y. (2018). Effect of a synthetic nano-CaO-Al_2_O_3_-SiO_2_-H_2_O Gel on the early-stage shrinkage performance of alkali-activated sag mortars. Materials.

[74] Palacios M., Puertas F. (2005). Effect of superplasticizer and shrinkage-reducing admixtures on alkali-activated slag pastes and mortars. Cem. Concr. Res..

[75] Zhang W., Lin H., Xue M., Wang S., Ran J., Su F., Zhu J. (2022). Influence of shrinkage reducing admixtures on the performance of cementitious composites: A review. Constr. Build. Mater..

[76] Kalina L., Jr V.B., Novotny R. (2018). Influence of alkali ions on the efficiency of shrinkage reduction by polypropylene glycol in alkali activated systems. Adv. Cem. Res..

[77] Hu X., Shi C.J., Zhang Z.H., Hu Z.L. (2019). Autogenous and drying shrinkage of alkali activated slag mortars. J. Am. Ceram. Soc..

[78] Yang Z., Shi P., Zhang Y., Li Z. (2022). Effect of superabsorbent polymer introduction on properties of alkali-activated slag mortar. Constr. Build. Mater..

[79] Song C., Choi Y.C., Choi S. (2016). Effect of internal curing by superabsorbent polymers-Internal relative humidity and autogenous shrinkage of alkali activated slag mortars. Constr. Build. Mater..

[80] Xiang J., He Y., Liu L., Zheng H., Cui X. (2020). Exothermic behavior and drying shrinkage of alkali-activated slag concrete by low temperature-preparation method. Constr. Build. Mater..

[81] Thomas R.J., Lezama D., Peethamparan S. (2017). On drying shrinkage in alkali-activated concrete: Improving dimensional stability by aging or heat-curing. Cem. Concr. Res..

[82] Chi M.C., Chang J.J., Huang R. (2012). Strength and drying shrinkage of alkali-activated slag paste and mortar. Adv. Civ. Eng..

[83] Adesanya E., Ohenoja K., Kinnunen P., Illikainen M. (2017). Properties and durability of alkali-activated ladle slag. Mater. Struct..

[84] Chen W., Xie Y., Li B., Li B., Wang J., Thom N. (2021). Role of aggregate and fibre in strength and drying shrinkage of alkali-activated slag mortar. Constr. Build. Mater..

[85] (2006). Standard Test Method for Sieve Analysis of Fine and Coarse Aggregates.

[86] (2009). Standard Test Method for Bulk Density (“Unit Weight”) and Voids in Aggregate.

[87] Appa Rao G. (2001). Influence of silica fume on long-term strength of mortars containing different aggregate fractions. Cem. Concr. Res..

[88] Ou Z., Feng R., Li F., Liu G., Li N. (2022). Development of drying shrinkage model for alkali-activated slag concrete. Constr. Build. Mater..

[89] (2014). Standard Practice for Mechanical Mixing of Hydraulic Cement Pastes and Mortars of Plastic Consistency.

[90] (2008). Standard Specification for Flow Table for Use in Tests of Hydraulic Cement.

[91] (2011). Standard Test Method for Compressive Strength of Hydraulic Cement Mortars (Using 2-in. or [50-mm] Cube Specimens).

[92] (2011). Standard Practice for Use of Apparatus for the Determination of Length Change of Hardened Cement Paste, Mortar, and Concrete.

[93] (2021). Standard Test Method for Density, Absorption, and Voids in Hardened Concrete.

[94] Li L., Lu J., Shen P., Sun K., Pua L.E.L., Xiao J., Poon C.S. (2023). Roles of recycled fine aggregate and carbonated recycled fine aggregate in alkali-activated slag and glass powder mortar. Constr. Build. Mater..

[95] Haach V.G., Vasconcelos G., Lourenço P.B. (2011). Influence of aggregates grading and water/cement ratio in workability and hardened properties of mortars. Constr. Build. Mater..

[96] Khan M.N.N., Sarker P.K. (2020). Effect of waste glass fine aggregate on the strength, durability and high temperature resistance of alkali-activated fly ash and GGBFS blended mortar. Constr. Build. Mater..

[97] Gholampour A., Ho V.D., Ozbakkaloglu T. (2019). Ambient-cured geopolymer mortars prepared with waste-based sands: Mechanical and durability-related properties and microstructure. Compos. Part B Eng..

[98] Shettima A.U., Hussin M.W., Ahmad Y., Mirza J. (2016). Evaluation of iron ore tailings as replacement for fine aggregate in concrete. Constr. Build. Mater..

[99] Wang S., Wu K., Yang Z., Tang L. (2023). Long-term (2 years) drying shrinkage evaluation of alkali-activated slag mortar: Experiments and partial factor analysis. Case Stud. Constr. Mater..

[100] Chi M. (2012). Effects of dosage of alkali-activated solution and curing conditions on the properties and durability of alkali-activated slag concrete. Constr. Build. Mater..

[101] Steinerova M. (2011). Mechanical properties of geopolymer mortars in relation to their porous structure. Ceram.—Silik..

[102] Bilim C., Karahan O., Atiş C.D., Ilkentapar S. (2013). Influence of admixtures on the properties of alkali-activated slag mortars subjected to different curing conditions. Mater. Des..

[103] Tsiskreli G.D., Dzhavakhidze A.N. (1970). The effect of aggregate size on strength and deformation of concrete. Hydrotech. Constr..

[104] Lyu K., She W., Chang H., Gu Y. (2020). Effect of fine aggregate size on the overlapping of interfacial transition zone (ITZ) in mortars. Constr. Build. Mater..

[105] Tu Y., Yu H., Ma H., Han W., Diao Y. (2022). Experimental study of the relationship between bond strength of aggregates interface and microhardness of ITZ in concrete. Constr. Build. Mater..

[106] Zhang H., Ji T., Liu H. (2019). Performance evolution of the interfacial transition zone (ITZ) in recycled aggregate concrete under external sulfate attacks and dry-wet cycling. Constr. Build. Mater..

[107] Živica V. (2007). Effects of type and dosage of alkaline activator and temperature on the properties of alkali-activated slag mixtures. Constr. Build. Mater..

[108] Karagüler M.E., Yatağan M.S. (2018). Effect of aggregate size on the restrained shrinkage of the concrete and mortar. MOJ Civ. Eng..

[109] Yang T., Zhu H., Zhang Z. (2017). Influence of fly ash on the pore structure and shrinkage characteristics of metakaolin-based geopolymer pastes and mortars. Constr. Build. Mater..

[110] Zhao H., Li W., Gan Y., Wang K., Luo Z. (2023). Nano/microcharacterization and image analysis on bonding behaviour of ITZs in recycled concrete enhanced with waste glass powder. Constr. Build. Mater..

[111] Lyu K., Sun B., Liu X., Xie X., Liu R. (2022). Evaluation of the ITZ modification efficiency via aggregate surface coating with nano SiO_2_ (NS) and its influence on properties. Case Stud. Constr. Mater..

[112] Aghajanian A., Cimentada A., Fayyaz M., Brand A.S., Thomas C. (2023). ITZ microanalysis of cement-based building materials with incorporation of siderurgical aggregates. J. Build. Eng..

